# Hydrophenoxylation of alkynes by gold catalysts: a mini review

**DOI:** 10.1007/s00894-024-06152-3

**Published:** 2024-09-30

**Authors:** Miguel Ramos, Miquel Solà, Albert Poater

**Affiliations:** Institut de Química Computacional i Catàlisi and Departament de Química, Universitat de Girona, c/Maria Aurèlia Capmany 69, 17003 Girona, Catalonia Spain

**Keywords:** Hydrophenoxylation, Alkyne, Dual metal catalysis, Gold

## Abstract

**Context:**

The field of chemistry has significantly evolved, with catalysis playing a crucial role in transforming chemical processes. From Valerius’ use of sulfuric acid in the sixteenth century to modern advancements, catalysis has driven innovations across various industries. The introduction of gold as a catalyst marked a pivotal shift, expanding its applications beyond ornamentation to homogeneous catalysis. Gold’s unique properties, such as its electrophilic nature and flexibility, have enabled its use in synthesizing complex molecules, including those in nanomedicine and sustainable chemical processes. The development of gold-based complexes, particularly in hydroalkoxylation and hydroamination reactions, showcases their efficiency in forming carbon–oxygen bonds under mild conditions. Recent studies on dual gold catalysis and heterobimetallic complexes further highlight gold’s versatility in achieving high turnover rates and selectivity. This evolution underscores the potential of gold catalysis in advancing environmentally sustainable methodologies and enhancing the scope of modern synthetic chemistry. The debate about the nature of monogold and dual-gold catalysis is open.

**Methods:**

DFT calculations have played a key role in promoting the activation of alkynes, in particular the hydrophenoxylation of alkynes by metal-based catalysts. They not only help identify the most efficient and selective catalysts but also aid in screening for those capable of performing a dual metal catalytic mechanism. The most commonly used functionals are BP86 and B3LYP, with the SVP and 6-31G(d) basis sets employed for geometry optimizations, and M06 with TZVP or 6-311G(d,p) basis sets used for single-point energy calculations in a solvent. Grimme dispersion correction has been explicitly added either in the solvent single point energy calculations or in the gas phase geometry optimizations or in both. To point out that M06 implicitly includes part of this dispersion scheme.

## Introduction

Over time, the field of chemistry has continually evolved, seeking deeper insights into chemical transformations to achieve better control over them. In the sixteenth century, Valerius utilized sulfuric acid to catalyze the conversion of alcohol into ether and amylase. By 1833, sulfuric acid was also recognized as a catalyst for the formation of starch sugar. A few years later, Berzelius introduced the term catalysis, formalizing a field dedicated to enhancing reaction rates through the use of specific species, now known as catalysts [1].

The use of metals in catalysis developed concurrently with advancements in heterogeneous catalysis. An early example is the oxidation of alcohol to acetic acid on platinum when exposed to air [2]. Subsequent studies explored other metals such as molybdenum, nickel, and silver. The nineteenth century saw significant progress in catalysis, culminating in the modern chemical and petrochemical industries, highlighted by Eugene Jules Houdry’s catalytic cracking process. Additionally, the invention of “celluloid” in 1860 marked the emergence of the first synthetic plastic-like polymer, addressing the shortage of natural materials such as ivory and tortoiseshell [3].

Nowadays, the majority of materials are produced through catalysis, which enables the creation of essential goods such as medicines and much more. This process forms one of the pillars supporting today’s society. Plastics, or more broadly, polymers, are crucial products that replace natural materials. However, with the well-being of the Earth at stake, it is necessary to develop processes and explore ways to make these materials less harmful to the environment. Gold, once primarily known for its ornamental value, has evolved significantly over the past half-century [4]. It has become a key player in homogenous catalysis [5–9], expanding its applications in both reactivity [10, 11] and sustainability [12], and additional ones are anticipated to reach the industrial market [13].

## Catalysis in gold chemistry

The dawn of gold catalysis emerged when Bond et al. provided experimental evidence of a bulk gold catalyst performing olefin hydrogenation [14]. This discovery ignited a surge of research aimed at understanding and enhancing the potential of gold chemistry, once regarded as an inert metal due to its stability [15]. Today, gold’s applications extend beyond catalysis. Its remarkable flexibility makes it valuable for constructing nanoparticles used in nanomedicine [16], particularly in cancer treatment, due to its strong and tuneable optical properties [17]. Additionally, gold’s role in heterogeneous catalysis remains a significant focus for chemists and materials scientists [18].

**Fig. 1:**
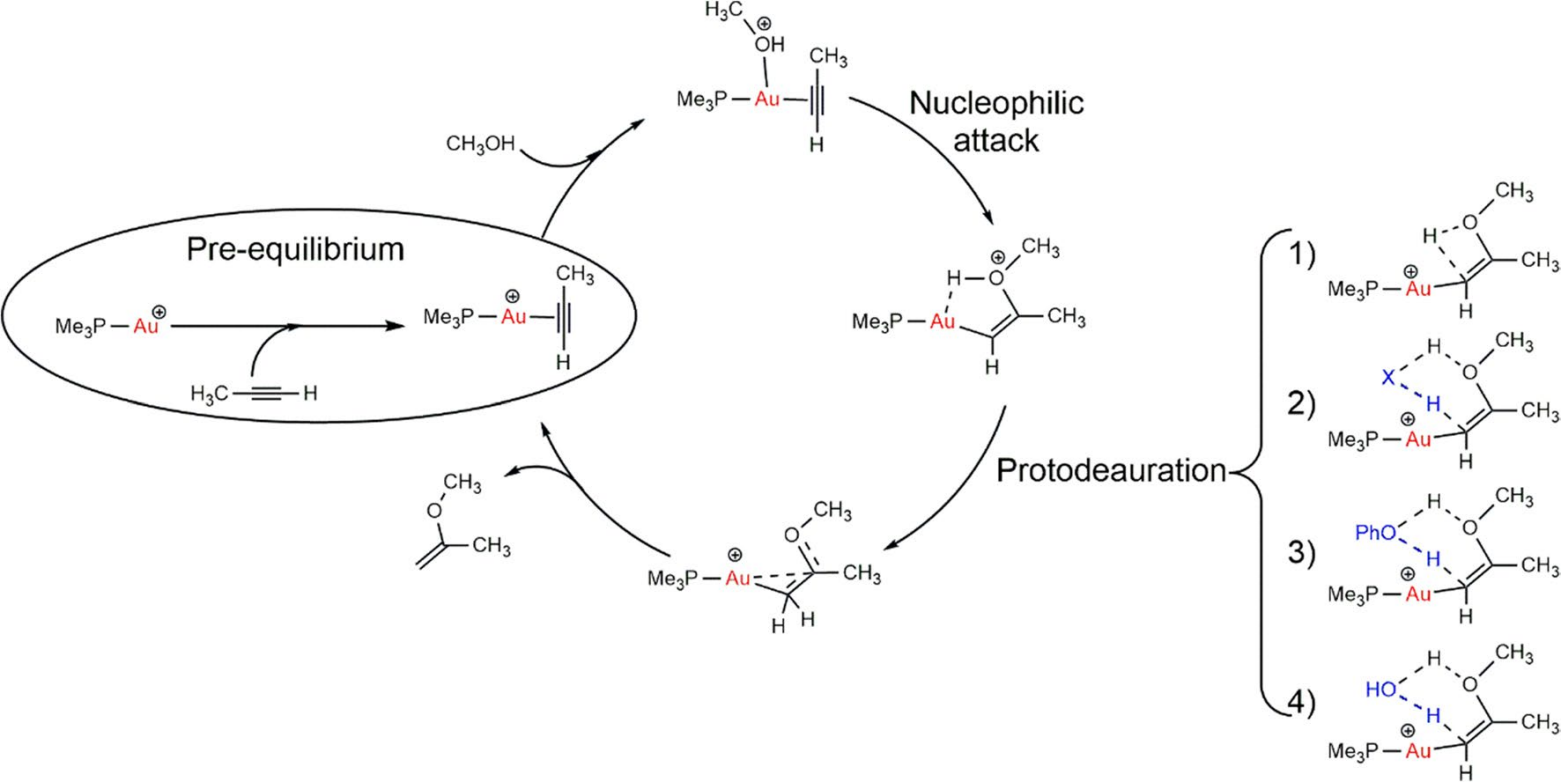
Proposed catalytic cycle for vinyl ether synthesis with methanol and propyne by phosphinegold(I) catalyst, including four possible scenarios of the protodeauration step

Equally notable is the efficiency and richness of gold in homogeneous catalysis. The initial phase of using gold in this area involved cationic linear species of gold(I) with phosphine ligands [19] and gold(III) salts [20]. In 1998, Teles et al. proposed using cationic phosphinegold(I) instead of zinc silicates [21] or the toxic mercury(II) salts for the addition of alcohols to alkynes [22]. This reaction successfully activates molecules with unsaturated systems, achieving affordable and competitive conversion rates with high turnover numbers (TONs) and turnover frequencies (TOFs). However, it requires substantial amounts of strong acid relative to the catalyst loading. Teles et al. also proposed a gold-alkyne activation mechanism supported by ab initio calculations (Fig. 1).

Among the various capabilities of gold(I) complexes, the activation of alkynes stands out as particularly significant [23–27]. This is primarily due to gold’s soft metal nature and its electrophilic character, which makes it a remarkable electropositive metal with a notable ability to accept electrons. When gold activates alkynes, it makes them highly susceptible to nucleophilic attacks, even from moderately weak nucleophiles, because the alkyne’s LUMO, which typically has low energy, becomes even more accessible [28].

**Fig. 2:**
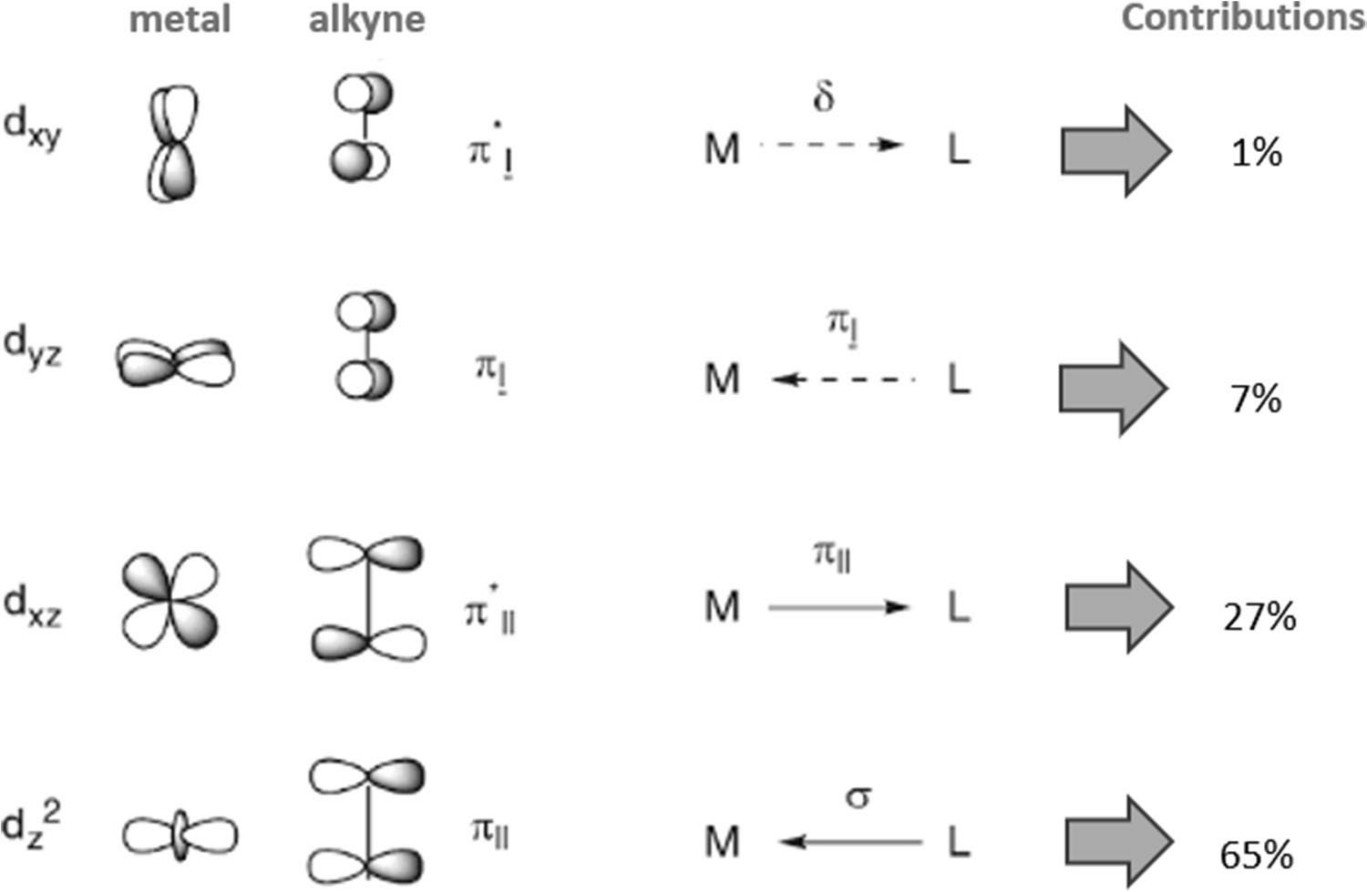
Qualitative contributions between Au^I^ and alkynes. Low lying LUMO of alkynes interacts with d-orbitals of metal (gold) to result in π-bond [30]

As illustrated in Fig. 2, the interaction between gold(I) and alkynes involves four main contributions based on the orbital symmetries for a d^10^-gold complex. The σ-contribution accounts for more than half of the total bonding interaction, making it the most significant factor. The in-plane π_||_ back donation is the second most important contribution. In contrast, the orthogonal π_⊥_ and δ overlapping symmetry terms contribute weakly. Consequently, alkynes act as strong two-electron *σ* donors but are less inclined to accept *π* electrons from gold(I), although some degree of back donation does occur [29].

The addition of alcohols to alkynes, known as hydroalkoxylation reactions, has garnered significant interest in the field of gold catalysis [31]. This type of reaction is particularly intriguing due to gold’s unique ability to activate alkynes, facilitating the formation of carbon–oxygen bonds with high efficiency and selectivity. Actually, hydroalkoxylation reactions catalyzed by gold offer several advantages. They often proceed under mild conditions, reducing the need for harsh reagents or high temperatures, and typically exhibit excellent functional group tolerance, allowing for the synthesis of complex molecules with high precision. Moreover, these reactions can be highly regio- and stereoselective, making them valuable tools in the synthesis of fine chemicals, pharmaceuticals, and natural products. In recent years, research has focused on optimizing gold catalysts to improve their activity and selectivity in hydroalkoxylation reactions. This includes the development of various ligand systems that can enhance the catalytic performance of gold complexes. Additionally, mechanistic studies have provided deeper insights into how gold activates alkynes and facilitates the addition of alcohols, paving the way for the design of more efficient catalytic systems.

Overall, the exploration of hydroalkoxylation reactions in gold catalysis continues to be a vibrant area of research, offering promising prospects for the advancement of organic synthesis and the development of new methodologies for constructing valuable molecular architectures.

## Hydroalkoxylation and hydration of alkynes

In general, homogeneous gold(I) catalysis involves complexes with two open sites in the coordination sphere. Typically, one of these sites is occupied by a labile ligand, while the other is occupied by a stable ligand that is strongly bonded to the metal [32]. Historically, this stable ligand has predominantly been phosphine-based. In 2008, Nolan and coworkers introduced a gold-based complex with N-heterocyclic carbene (NHC) ligands, such as IPr (1,3-bis(2,6-diphenylmethyl)imidazol-2-ylidene). This complex facilitated the gold(I) hydration reaction under mild conditions and with low catalyst loading, marking a significant improvement in catalytic activity [33]. Additionally, concurrent reports highlighted the crucial role of silver(I) salts in activating gold catalysts for both di- and monogold complexes (Eq. 1) [34–36]. These studies also underscored the importance of the counter-ion in stabilizing intermediates [37], a topic on which Belanzoni, Zucaccia, and their coworkers have made substantial contributions [38–40].

1 $$\underset{\mathrm X=\mathrm{NTf}_2^-,\mathrm{OTf}^-,\mathrm{OCl}_4^-\mathrm{BF}_4^-,\mathrm{PF}_6^-,\mathrm{SbF}_6^-}{\mathrm L-\mathrm{Au}\cdot\mathrm{CI}\;\;\xrightarrow{\mathrm{AgX}}\;\mathrm{AgCI}\;+\;\mathrm L-\mathrm{Au}-\mathrm X}$$

**Fig. 3:**
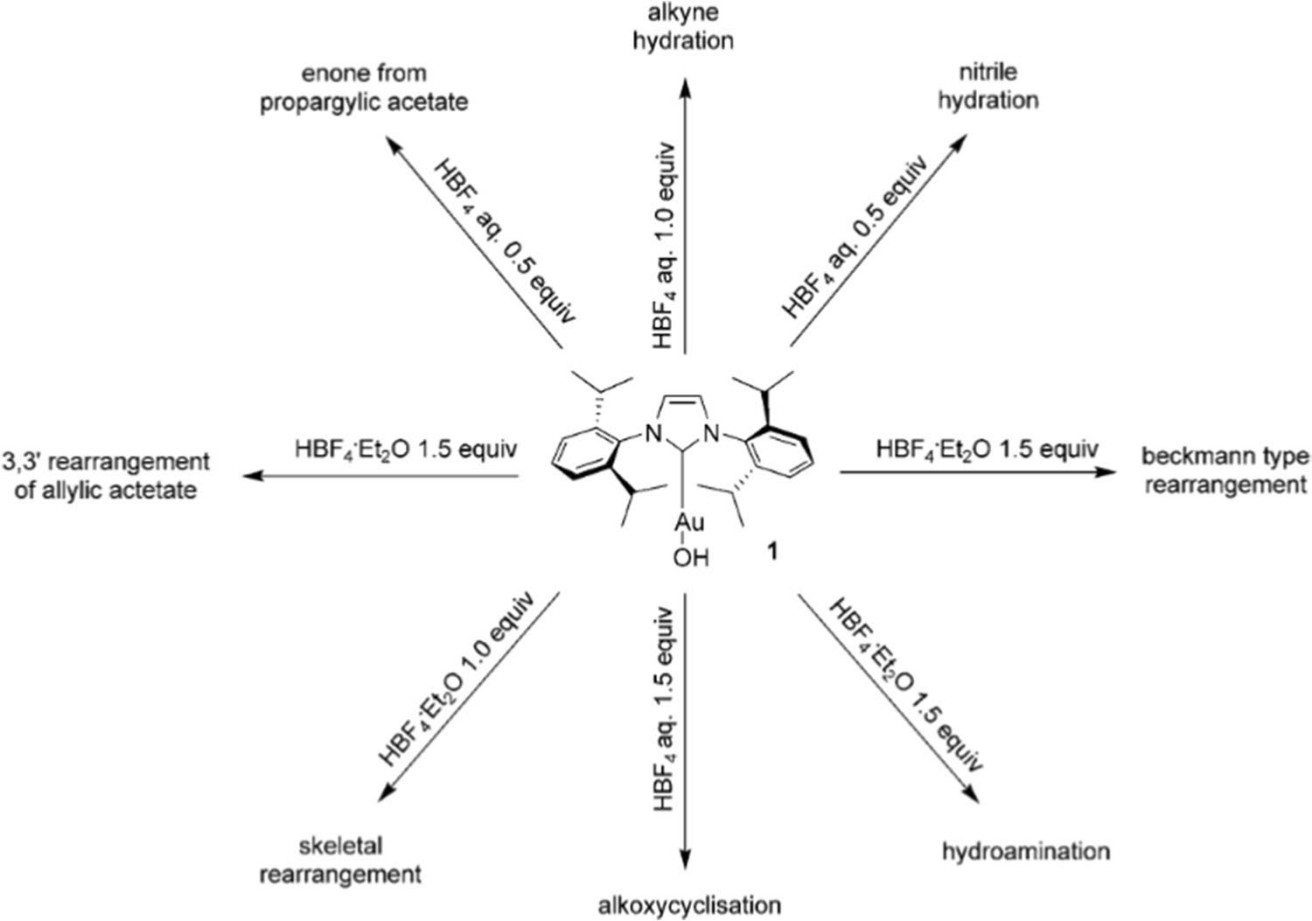
Precursor of [Au(IPr)][BF_4_] stable electrophile and some of the reaction routes that require the alkyne activation [48]

It has been observed in hydroalkoxylation reactions mediated by gold catalysts that the bulkiness of the ligand is crucial for achieving long catalyst lifetimes [41]. Consequently, complexes bearing NHC-ligands are of significant interest in the field of gold(I) catalysis. These ligands allow for the modulation and adaptation of not only the steric [42], but also the electronic properties of the metal complex, directly affecting the catalyst’s reactivity and stability [43]. Various research groups have made substantial efforts to accurately model the bonding of these species and optimize the ligand effects [44, 45]. The interaction of NHC-gold(I) catalysts with alkynes forms π-complexes with a high electrophilic character, capable of readily reacting with alcohols or amines to produce vinyl ethers or amines [46]. However, it has been shown that large NHC-gold(I) complexes generally have lower efficiency in the hydroalkoxylation of propargylic alcohol [47]. Additionally, the activation of these complexes does not require the application of silver(I) salts, making them a valuable synthetic tool (Fig. 3).

Hydroalkoxylation and hydration reactions have been successfully applied in synthesis to construct C-O bonds from olefins [49], allenes [50], and alkynes, producing vinyl ethers and other products [51]. The intermolecular hydrophenoxylation of internal alkynes to synthesize vinyl ethers has long been a topic of interest [52]. Notably, Hashmi and coworkers have reported excellent turnover numbers (TONs) in gold(I)-catalyzed hydroalkoxylation of activated alkynes [41, 53]. Despite being known as entropically unfavorable reactions, significant efforts have been made to advance this field. Kuram et al. reported the hydrophenoxylation of symmetrical and non-symmetrical alkynes using a gold(III) catalyst in the presence of a mild or strong base [54].

To support the concept of gold activation, in silico predictions have been crucial in complementing experimental results [9, 55–57], and even more when calculations anticipate selective experiments [42, 58]. To explore the rate-determining step (rds) in the reaction, Pernpointner and coworkers conducted computational studies on the mechanism of phenol addition to alkenes catalyzed by phosphine-gold(I) [59], previously investigated by Ujaque and coworkers [60]. These studies indicated an active role of water and phenol in the process. Determining the precise mechanics of the RDS was challenging, with uncertainty surrounding the final protonation that releases the product [57]. Additionally, how this final step proceeds is under debate, leading to the suggestion of four possible scenarios for the protodeauration step (Fig. 1): (1) direct proton transfer, (2) proton transfer assisted by the counter-ion, (3) proton transfer assisted by phenol, and (4) proton transfer assisted by water. It was found that the pathway promoted by either water or phenol proceeds in a concerted manner, while the other two scenarios were unfeasible due to their higher energy barriers (61.1 and 49.6 kcal/mol, respectively, compared to pathways 1 and 2).

## Is it possible to move beyond the monometal approach?

Despite extensive computational efforts to understand dual metal catalysis in hydrophenoxylation reactions, as will be detailed below, there remains a need for a qualitative description of the mechanism of C-O bond formation, in particular the interaction between the metal and the ligand/s [61]. Most studies to date focus on single gold catalysis. Notably, various investigations have explored both the thermodynamics and kinetics of these processes [62]. In 2004, Nemcsok, Meyer, and colleagues examined the interactions between NHC ligands and group 11 metals [63, 64], shedding light on the importance and nature of these interactions. More recently, a study provided insights into the hydroamination reaction, specifically analyzing the C-N bond formation between an alkyne and an amine catalyzed by rhodium [65, 66] and gold [67–69].

## Dual gold catalysis

**Fig. 4:**
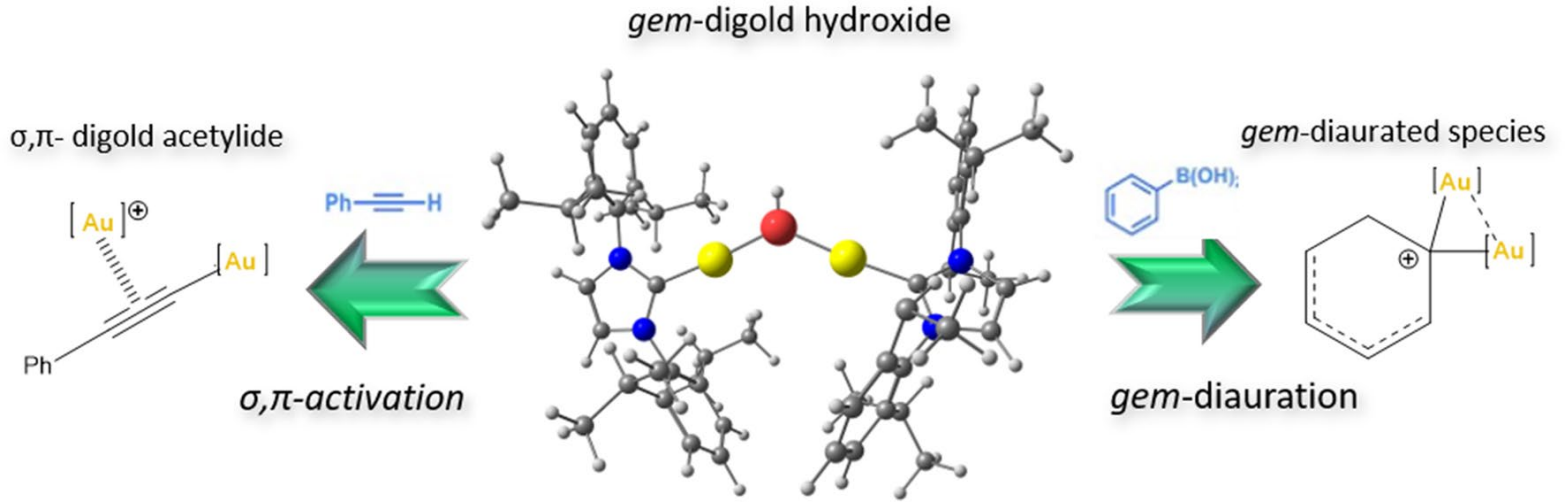
Usual precursor in dual gold activation catalysis [{Au(NHC)}_2_(μ-OH)](*gem*-digold hydroxide) ([Au] = Au(NHC)) [76]

In 2008, Houk and Toste and coworkers shed light into gold catalysis, providing the experience of two gold centers forming a diaurated complex synergistically cooperating to make a reaction of cycloisomerization [70]. In that report, the authors explained the mechanism to proceed through the activation of an in situ–formed phosphinegold(I) acetylide by cationic phosphinegold(I). One year later, Gagosz’s group studied a similar reactivity for the phosphinegold(I) catalyst highlighting its unusual dual character as nucleophile (C-H activation) and electrophile (alkyne activation) [71]. These first discoveries of cyclization reactions with allenyne and dyines molecules, which contain C–C triple bond, enabled gold to form the later called σ,π-digold-acetylide complex [72–74], which marks the beginning of this new special synthesis together with the stable *gem*-diaurated species (Fig. 4), even with applications as anticancer agents [75].

*Gem*-diaurated species were first reported in 2003 by synthesizing thiophenes with the reaction of monogold organometallic compounds and stoichiometric amounts of a cationic phosphine-gold complex [77]. The synthesis of complexes with two gold centers located next to each other is highly efficient and allows the activation of the reagents; however, despite the relevance of the discovery, these species were only considered a catalyst reservoir not involved in the product-yielding steps [78]. Even if so, Nolan and coworkers have made relevant discoveries on these species trying to realize the real importance of *gem*-hydroxide and how it could be key to the catalysis of gold (I) [48, 79]. Finally, the exploration of the hydroalkoxylation mechanism by Roithová and coworkers proved that these species-mediated the addition of alcohols to alkynes [80, 81]. Another matter of fact is the extra-marked metallophilic behavior or aurophilicity in multi-gold compounds which adds stability for a large quantity of gold-based novel structures [82, 83].

**Fig. 5:**
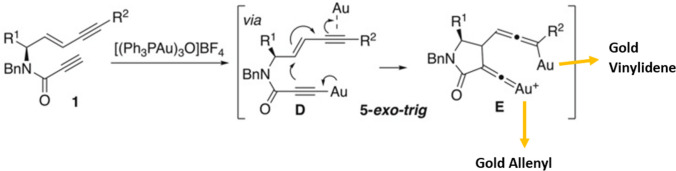
Formation step of usual intermediates from gold intramolecular transformations [87]

On the other hand, σ,π-digold-acetilyde species suggest assistance and cooperation from two equal gold atoms but different in their chemical behavior, whose formation is highly favored in the presence of alkynes [34, 73]. Furthermore, C(sp^3^)-H activated by gold may easily suffer cyclization through C-H insertion that involves the production of rather common gold vinylidene and gold allenyl molecules (Fig. 5) [84–86].

Dual gold complexes were rapidly seen very useful for the formation of C–C bonds involving aromatic systems and multiple C–C bonds [9, 88]. Additionally, C-N bond construction by hydroamination has been also thoroughly studied through new and energizing propositions for dual gold catalysis mechanisms [89–91], addressing important facts that help to understand the gold(I) chemistry and elucidate the role of these diaurated species in organometallic catalysis [92–95].

## Cooperative hydrophenoxylation of alkynes by gem-digold hydroxide catalyst

Regarding this recently discovered chemistry, *gem*-digold hydroxide specimens display the important role of highly efficient pre-catalysts in hydroalkoxylation of alkynes [48]. In this synthesis of (Z)-vinyl ethers, Nolan et al. studied the effective catalysis of bifunctional gold that forms the intermolecular C-O bond between an alkyne and an alcohol. In this way, they obtained TON and TOF up to 35000 and 2188 h^−1^, respectively [96, 97].

In the presence of an alkyne, *gem*-digold hydroxides split into two separated fragments to perform a nucleophilic (combined) reaction. Herein cationic gold fragment acts as Lewis acid [98] and gold hydroxide acts as a Brønsted base (Eq. 2) that can deprotonate the alcohols to form alkoxides, which in that case phenoxide is mostly formed from phenol [99].
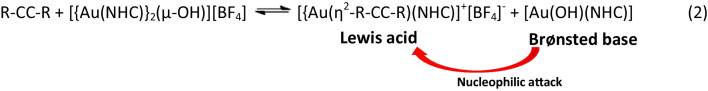


**Fig. 6:**
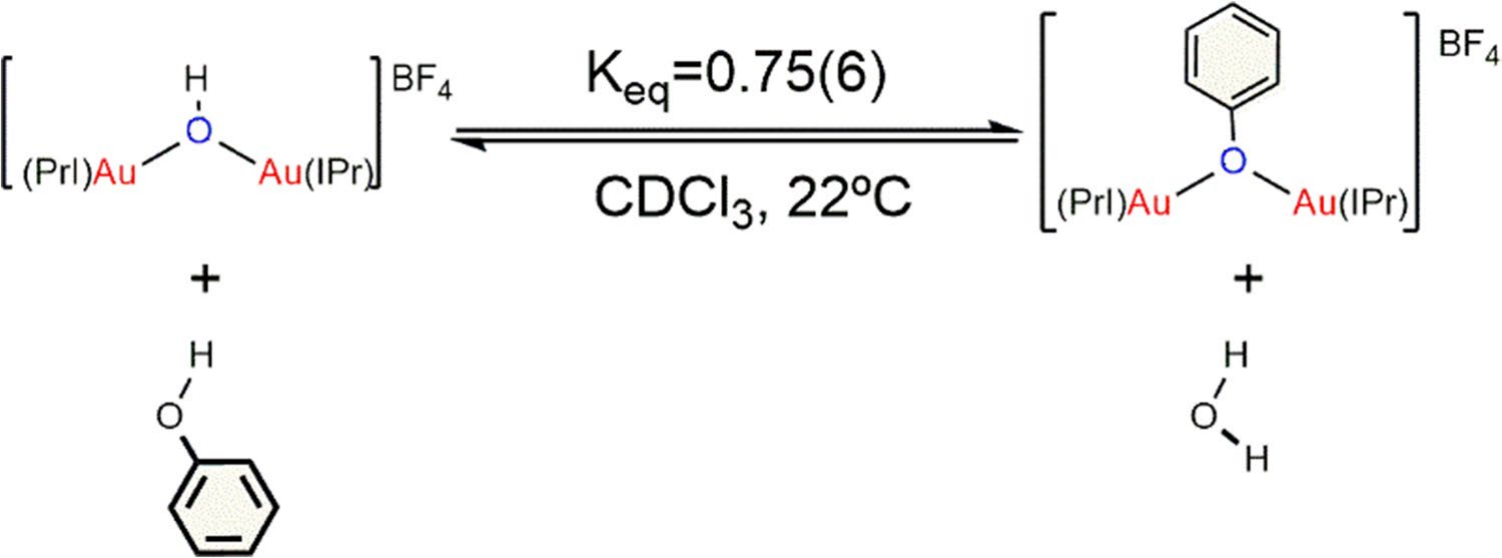
Equilibrium between *gem*-digold species

In this reaction, gold activation of substrates occurs separately and smoothly in an excess of alkyne, making the complexes more susceptible to interaction. This increased reactivity facilitates the nucleophilic attack of the phenoxide, which reacts in a specific manner to selectively produce *trans* vinyl ether monomers. Poater et al. conducted further studies to deepen the understanding of the mechanism for the hydrophenoxylation of alkynes in toluene [57]. Their initial findings confirmed the ability of gem-digold hydroxides to readily dissociate and re-associate (Fig. 6). They also observed the poor reactivity of phenol in monoaurated systems and highlighted the active role of water and the counter-ion in the reaction mechanism. Additionally, the reaction conditions ruled out water-mediated alkyne hydration over hydrophenoxylation, although water molecules were found to be crucial. Under anhydrous conditions, the reaction slows down significantly, indicating that water, in conjunction with phenol, plays a critical role in maintaining the balance of gem-digold species. These findings underscore the importance of water not only in facilitating the reaction but also in stabilizing the reactive intermediates. The insights provided by Poater et al. offer a comprehensive understanding of the nuanced roles of various components in the hydrophenoxylation of alkynes, paving the way for more efficient and selective catalytic processes.

**Fig. 7:**
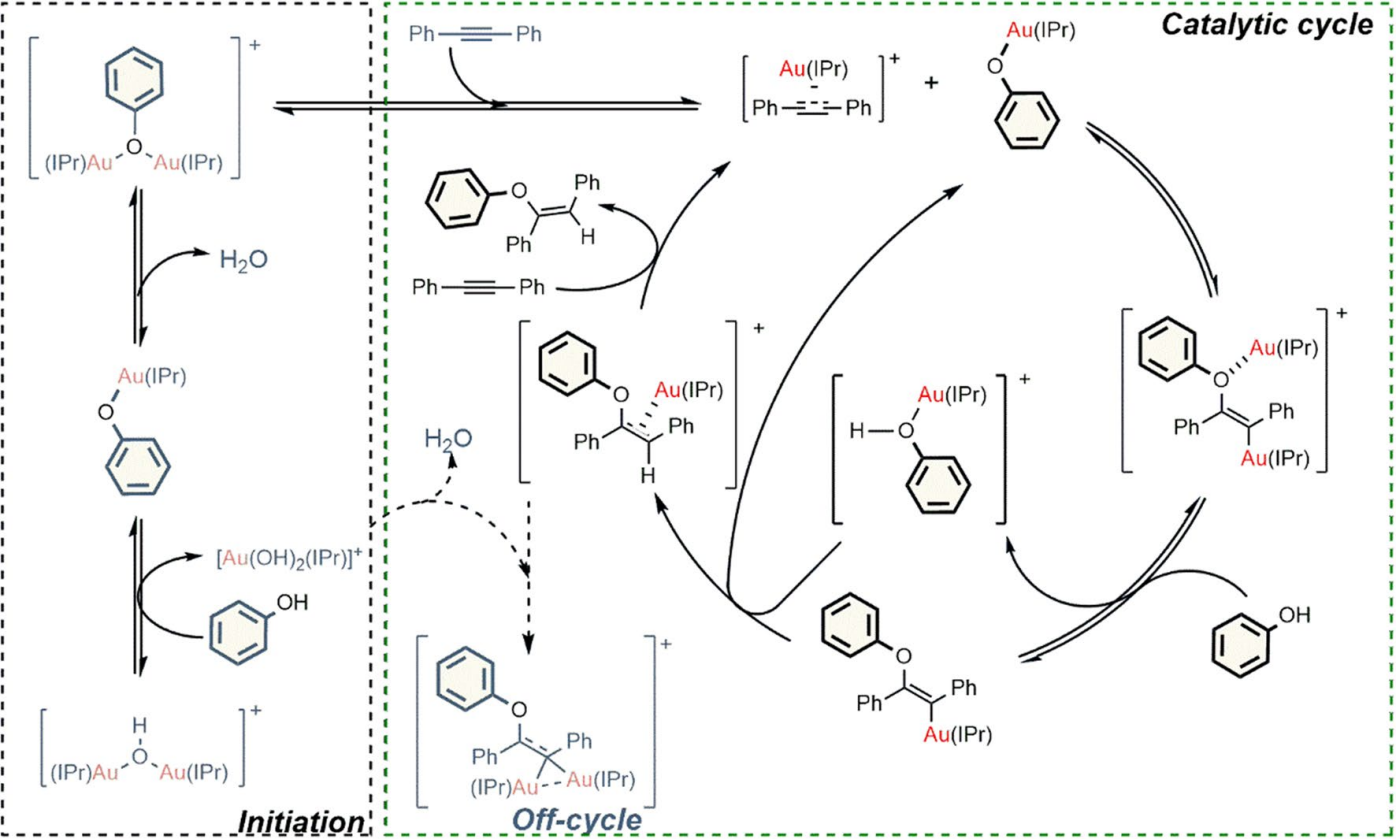
Hydrophenoxylation of alkynes by dual gold activation catalysis unveiled by DFT calculations [57]

Multiple possible mechanisms were studied to explore the total synthesis of vinyl ethers as there was evidence of assistance in the mechanism. All in all, first comes the activation of the two substrates by coordinating one gold center each other from *gem*-digold phenoxide; [{Au(NHC)}_2_(μ-OPh)], thus, at this point, one gold phenoxide [Au(OPh)(NHC)] and one gold alkyne [Au(NHC)(η^2^-alkyne)] spring out and the catalytic cycle begins (Fig. 7). Additionally, computational results with low potential energy *gem*-digold “off-cycle” species were revealed in this study [50].

Full conversion of *gem*-digold hydroxide to the aforementioned species is achieved and counter-ion is in charge of stabilizing the species. This nucleophilic attack from the phenoxide takes place and the C-O bond is formed. This step was reported by Zucaccia and coworkers to be the rds in the reaction of monogold [38] and actual studies made so for dually catalyzed mechanism. Subsequent protodeauration in the mechanism leads to the product and to the recovery of the catalyst.

To support the experimental data discussed above, density functional theory (DFT) calculations are crucial. They have been used for characterizing the stability and providing structural insights by identifying all intermediates along the reaction coordinate. They also found entropy to be particularly relevant during the C-O bond formation facilitated by the gold-activated substrates.

Further mechanistic studies on hydrophenoxylation by the group aimed to better understand the exact mechanism of dual gold-catalyzed hydrophenoxylation of alkynes and its advantages over the monogold mechanism [100, 101]. The effects of exchanging the NHC ligand for a less bulky one, such as 1,3-dimethylimidazol-2-ylidene (IMe), and using different substrates were also investigated.

**Fig. 8:**
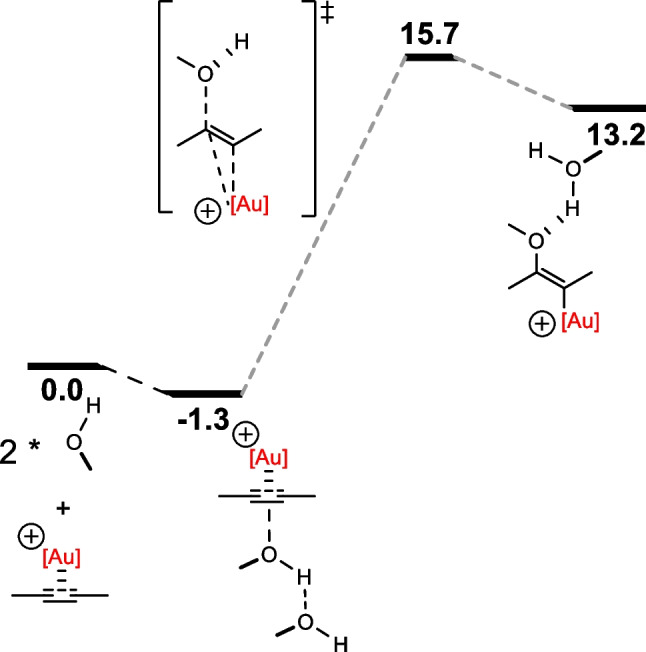
Relative Gibbs energies in the C-O bond formation reaction of methanol with [Au(IMe)(η^2^-Me-CC-Me)]^+^, where [Au] = [Au(IMe)]

They began by energetically defining the rds for single auration of the alkyne. Results suggested that the direct nucleophilic attack by free alcohol is highly dependent on whether the alcohol is alkylic or arylic and requires the assistance of the counter-anion or a co-catalyst, as it is neither kinetically nor thermodynamically favorable (Fig. 8).

**Fig. 9:**
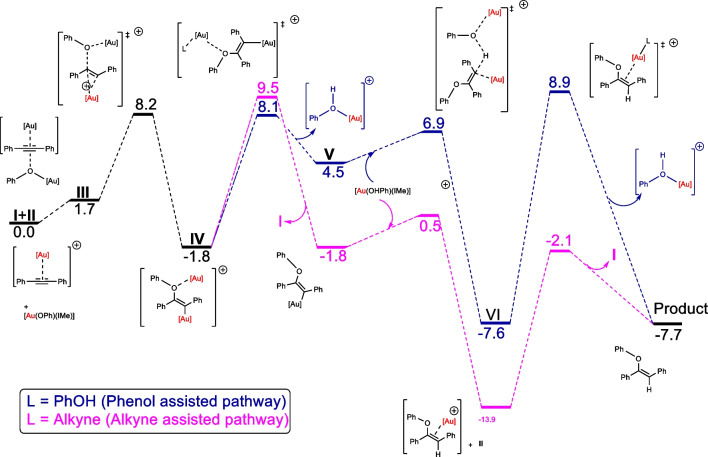
Hydrophenoxylation of diphenylacetylene mechanism Gibbs energies computationally defined ([Au] = Au(NHC)). Theory level: M06/TZVP∼sdd(toluene)//BP86/SVP∼sdd

In the framework of dual activation applied in the hydrophenoxylation of alkynes, the reaction proceeds mildly and with low catalyst loadings. Using DFT to define the Gibbs energies for the process, assistance from substrates was seen to be of high relevance in the process (Fig. 9). At the C-O bond formation step, dual gold–catalyzed hydrophenoxylation of diphenylacetylene mechanism left out of the game that of the mono-catalyzed one, having two phenols to cooperatively conduct the nucleophilic attack, by displaying a much lower energy barrier (ΔG^‡^).

In general, lower ΔG^‡^ are achieved directly affecting the rds. Activation of acidic phenol alcohol improving the selectivity and reactivity sets dual gold catalysis at a whole new level from that of mononuclear gold hydrophenoxylation of alkynes, as it is thermodynamically favorable, even once the entropic penalty of this bimolecular reaction is included. However, to obtain the *cis* vinyl ether product, it was also claimed that the dual catalysis was not convenient, and, for this purpose, monogold catalysis may achieve the feat.

Cooperative catalysis using a linear gold(I) complex that has dual behavior far surpasses that of the monogold in terms of reactivity and selectivity as to answer unequivocally the question “Why dual gold catalysis over mono gold?” whose response is summarized in two points:

- Broaden the possible coordination sphere and the scope of the reaction
- High-tier mechanism control by activation of both substrates, lowering ΔG^‡^ thereof

The importance of this reaction lies in the rupture of the normal process, those that are more visible but not environmentally sustainable [102], to introduce sustainability by enabling a mild synthesis and high catalyst activity with a robust and versatile methodology [96]. Furthermore, Nolan et al. optimized dual catalysis, achieving greater TOF than previous reports in the field [99].

The mechanism for homodually aurated systems has been extensively studied, with efforts focused on optimizing the reaction pathway and establishing the key contributions. This includes understanding the role of the counter-ion and the assistance provided by various species in the reaction mixture. Through extensive research, the reaction pathway has been fine-tuned to improve efficiency and selectivity. The counter-ion has been identified as a crucial factor, stabilizing intermediates and facilitating key steps in the mechanism. Additionally, the assistance of other species present in the reaction mixture has been shown to significantly influence the overall process, enhancing the catalytic activity and ensuring a smoother progression of the reaction. These studies have provided valuable insights into the intricate dynamics of homodually aurated systems, laying the groundwork for further advancements in the field of gold catalysis.

## Heterodual bimetallic cooperative catalysis

Inspired by the complexity and efficiency of multimetallic assemblies in enzyme catalysis, chemists have made significant strides in developing heterobimetallic complexes for applications in homogeneous catalysis [103]. These biological systems serve as a blueprint, highlighting how multiple metal centers can work in concert to enhance reactivity, selectivity, and efficiency. Building on this concept, researchers began by designing relatively simple heterobimetallic complexes that feature a combination of σ-donating and π-accepting ligands, such as N-heterocyclic carbenes (NHCs) and carbonyl ligands. Over the past two decades, these efforts have led to the development of increasingly sophisticated and diverse systems.

One of the primary advantages of heterobimetallic complexes over their monometallic counterparts is their potential to significantly boost catalytic performance. The presence of a second metal center in the active catalyst can open up entirely new reaction pathways, facilitating transformations that would be less efficient, or even impossible, with a single metal. This synergistic effect between the two metal centers allows for a more dynamic catalytic process, often resulting in increased reaction rates, improved selectivity, or access to more challenging substrates.

The mechanistic intricacies of heterobimetallic complexes in homogeneous catalysis can be explained by different factors. Take for instance, by examining how the second metal interacts with substrates, these complexes can be classified into distinct categories based on the nature of their interaction [103]. These interactions may range from simple electronic effects, such as the modulation of electron density between the two metals, to more complex cooperative behaviors where both metals actively participate in substrate activation and bond-forming steps.

Each class of heterobimetallic complex is illustrated with numerous examples, showcasing the vast range of catalytic reactions they can facilitate. These include traditional transformations like hydrogenation, hydrofunctionalization, and C–C bond formation, as well as more specialized processes that exploit the unique reactivity of bimetallic systems [103]. The examples underscore the flexibility and adaptability of heterobimetallic catalysts, which can be tailored to a variety of reaction conditions, substrates, and functional groups. Through these advances, heterobimetallic catalysis is poised to play an increasingly important role in the design of more efficient, sustainable, and selective catalytic processes in modern synthetic chemistry.

**Fig. 10:**
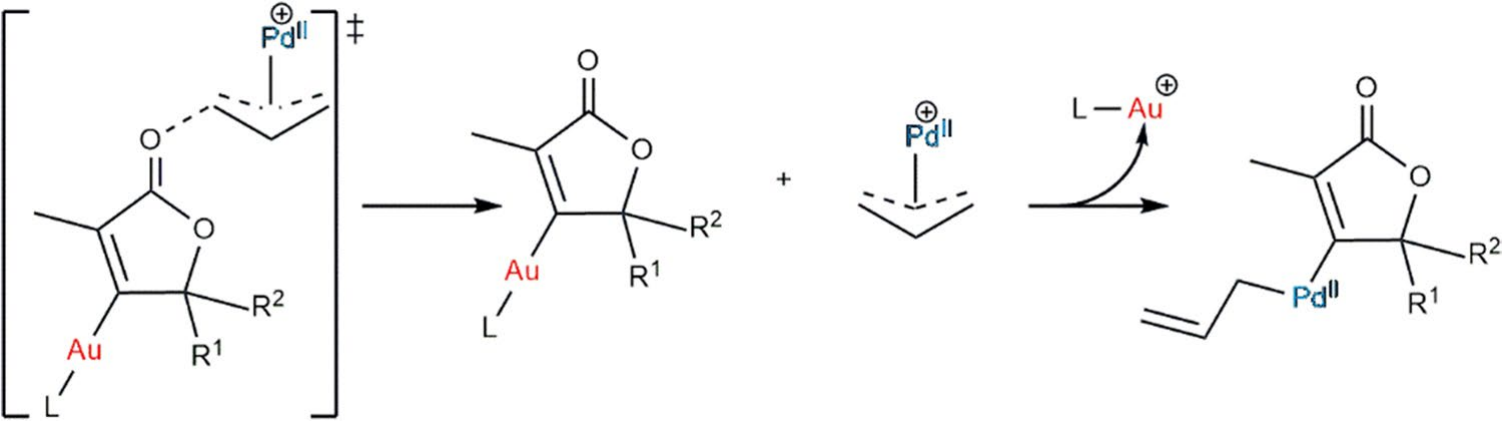
Simultaneous action of both metals: Activation with gold(I) Lewis acid lowers the barrier for palladium-catalyzed deallylation/oxidative addition [107]

Heterobimetallic complexes have attracted special interest due to the fact that the bifunctionality of different metals can lead to unique reactivity [104, 105]. Gold catalysis was early implemented with success in heterodual catalysis to conduct transmetallation and cross-coupling reactions together with rhodium, palladium, copper, and nickel to expand catalytic capabilities by surpassing the inherent complexity of dual systems [106]. Soft Lewis acidity of Au(I) was then seen to be of relevance in cooperative catalysis as it facilitated the way the reaction proceeds (Fig. 10).

Although there is actually some information about homogeneous heterodual bimetallic gold catalysis [108], it is mainly in heterogeneous catalysis where efforts have been more visible [109, 110]. To balance this scarcity, actual groups are progressing in the development of heterobimetallic complexes bearing one gold and another transition metal connected through a ligand. Low-valent palladium, zinc, and ruthenium, in addition to gold, bimetallic complexes, have been thoroughly studied for application into hydroarylation and hydroamination reactions as well [111, 112], but, due to the little separation between the metals, their utility seems limited to intramolecular transformations [113, 114].

Following the theme, recently Cazin et al. reported the hydrophenoxylation of internal alkynes with heterobimetallic Cu-NHC/Au-NHC systems. Here, the bifunctionality of the two metals is highlighted making the reaction proceed very smoothly [115]. Additionally, Au/Ag/Cu systems have been successfully applied in intramolecular cyclizations to synthetize heteroarenes [116–119].

**Fig. 11:**
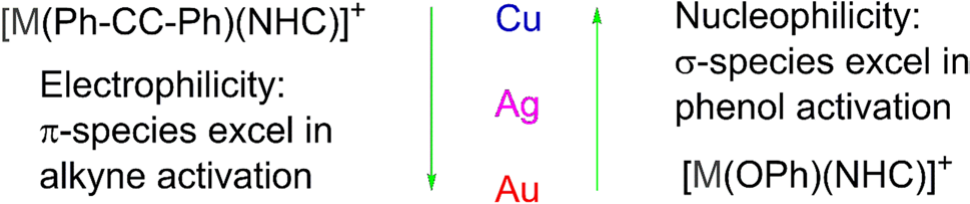
Natural bond order (NBO) character definition for group 11 metals

As a response to the search for improvements in the mechanism of hydrophenoxylation of alkynes, Poater and coworkers studied heterodual catalysis for group 11 metals by individually activating phenol and diphenylacetylene [120]. In this wide computational study, the authors characterized the tendency in the group for substrate activation, as the physicochemical differences between the three upper metals make them (Fig. 11) promote the C-O bond.

**Fig. 12:**
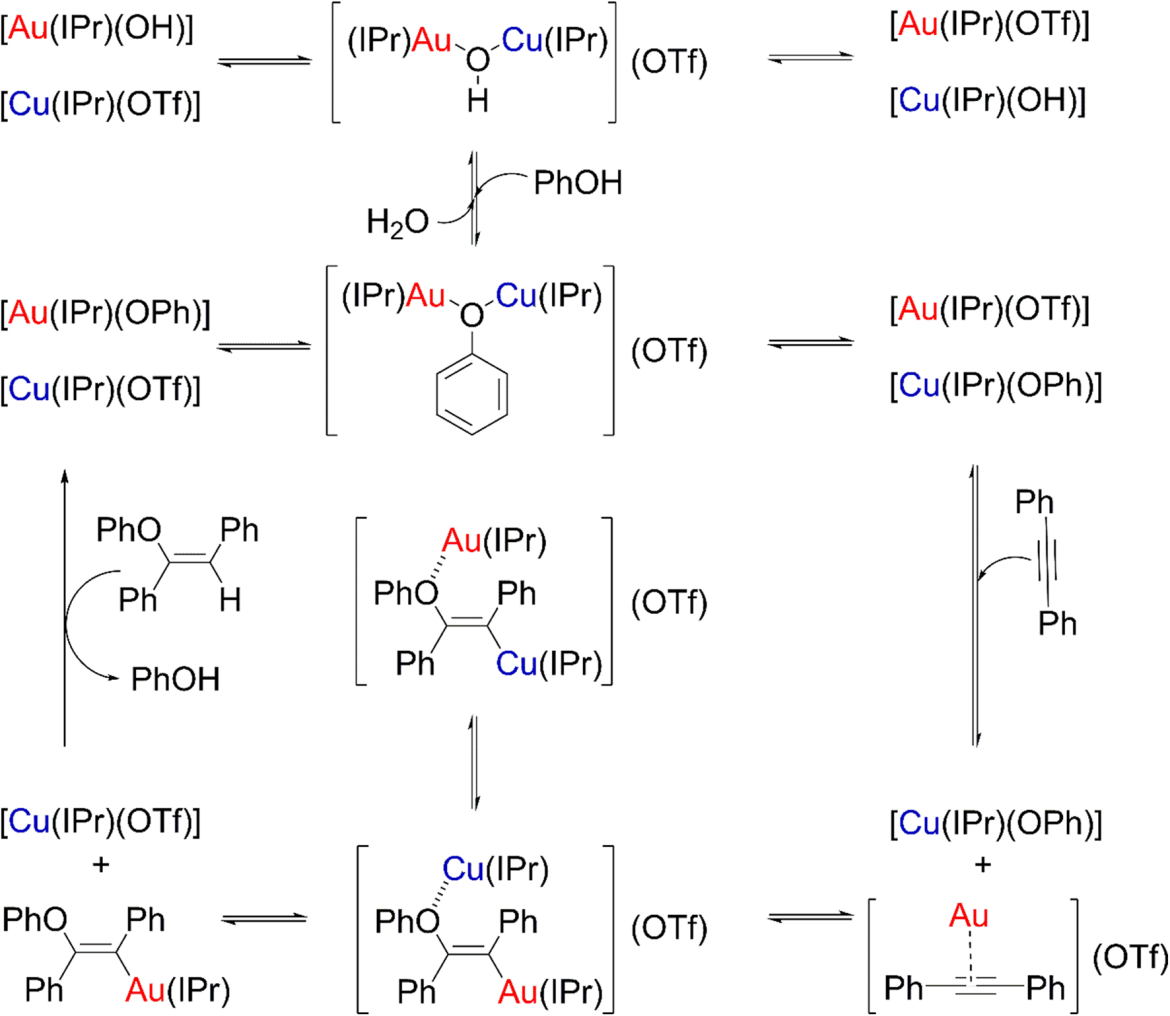
Mechanism for the hydrophenoxylation of internal alkynes assisted by gold/copper catalysts

First, gem-dimetal(I) hydroxides are the dimers which break to generate, through a series of balances and upon phenol and diphenylacetylene addition, the two aforementioned monometallic complexes. Second, the C-O bond is formed in a similar fashion as previously (Fig. 12) and after the transition state with both fragments approaching and the production of the intermediate.

**Fig. 13:**
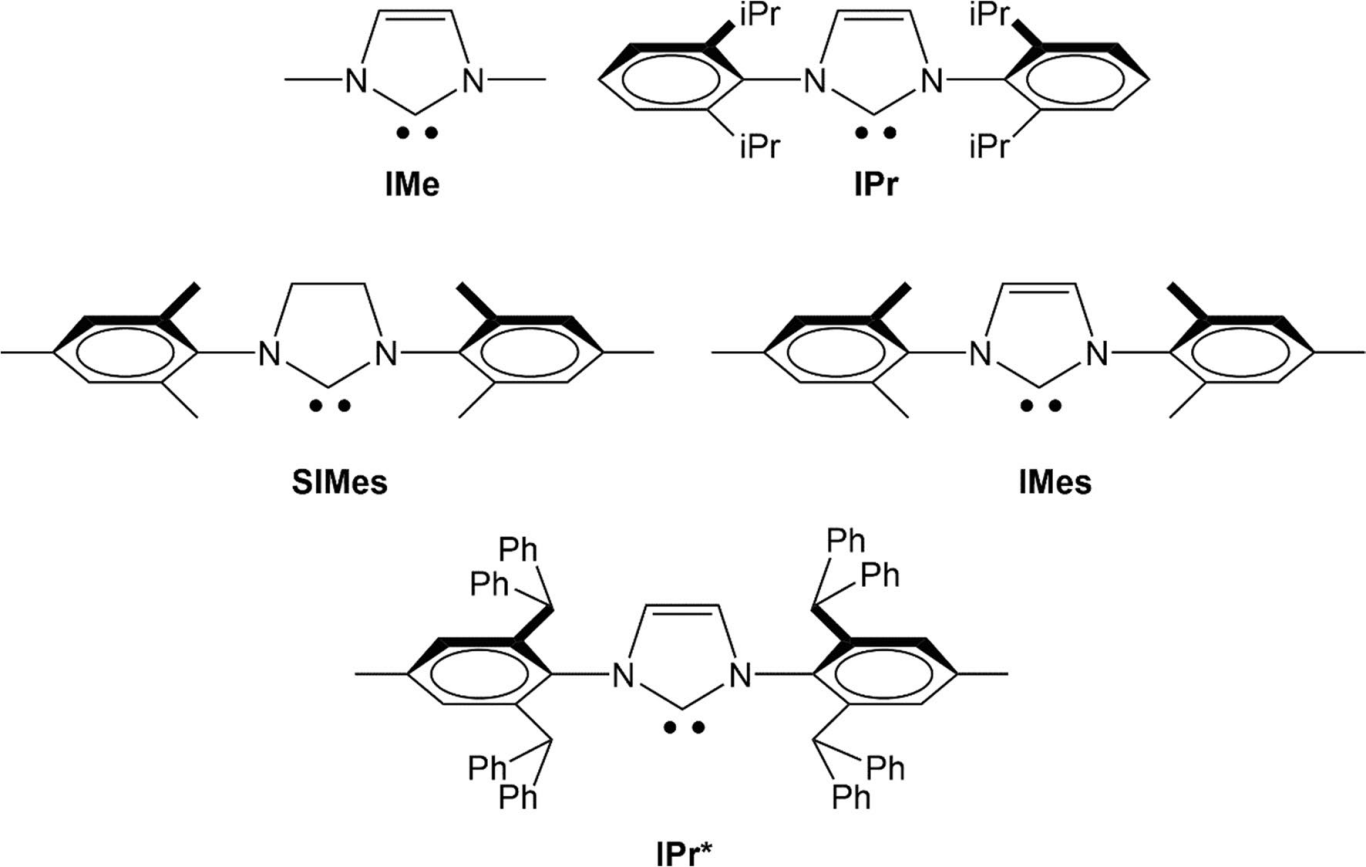
NHC involved in the steric and electronic studies of the rds

At the end, they showed the rds for six different bimetallic systems in order to see the inherent capacity of homobimetallic dual activation with respect to heterobimetallic process. Gold(I), as a better π acceptor, was seen to be very proficient in activating alkynes and making them more electrophilic. On the other hand, the chemical hardness of silver phenoxide was the lowest, and therefore, σ-silver species, together with π-gold species, showed more favorable energy barriers. Throughout the study, various NHC ligands are tested to see the relevance of the steric and electronic properties they play in the process of C-O bond formation (Fig. 13). In addition to IPr and IMe, they also explored medium-sized SiMes and IMes carbene ligands (SIMes = N,N′-bis(2,4,6-trimethylphenyl)-4,5-dihydroimidazol-2-ylidene) and (IMes = N,N′-bis(2,4,6-trimethylphenyl)imidazol-2-ylidene) and highly sterically hindered IPr* (IPr* = N,N′-bis(2,6-bis(diphenylmethyl)-4-methylphenyl)imidazol-2-ylidene).

In summary, the most efficient system identified for the hydrophenoxylation reaction was the π-Au/σ-Ag combination. However, it has been demonstrated that copper(I), which is more earth-abundant, can also efficiently activate phenol to facilitate the nucleophilic attack. This makes copper a more advantageous option due to its similar capacity for activation and greater abundance. Moreover, copper outperforms gold in the activation of alcohols, providing additional benefits for certain reactions.

Furthermore, the π-Au/σ-Cu mechanism has been thoroughly elucidated, offering deep insights into its operational dynamics. The studies revealed that the steric hindrance of NHC ligands significantly influences the reaction’s rds energy barriers. Specifically, it was found that medium-sized NHC ligands, such as IMes (1,3-dimesitylimidazol-2-ylidene) and SIMes (1,3-dimesitylimidazolidin-2-ylidene), present more favorable formation energies. This indicates that they strike a balance between steric bulk and electronic effects, optimizing the overall catalytic performance.

These findings underscore the potential of copper as a viable alternative to gold in catalysis, particularly in terms of cost-effectiveness and sustainability. They also highlight the importance of ligand design in tailoring the catalytic activity and efficiency of metal complexes in various reactions.

## Chelation of the complex to promote dual metal catalysis

**Fig. 14:**
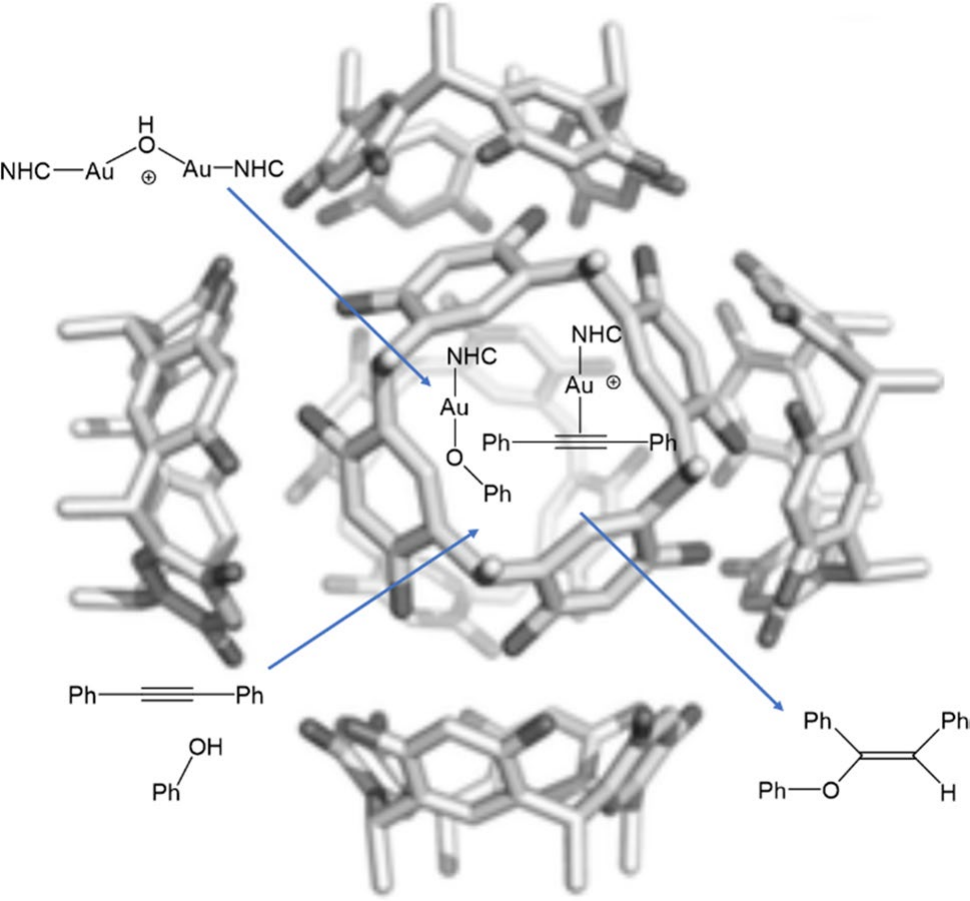
Encapsulated hydrophenoxylation of alkynes by [{Au(NHC)}_2_(μ-OH)]^+^

Recently, inspired by the encapsulation of a monogold(I) NHC complex reported by Reek and coworkers [121], Nolan et al. investigated the idea of encapsulating the diaurated NHC complex within a cavity to achieve switchable reactivity from dual gold catalysis to monogold catalysis [122], their sparked interest in the possibility of creating a chelate where two fragments, each containing a gold center, could be brought into close proximity by linking them with an ethylene chain (Fig. 14). This arrangement is expected to enhance process activity and selectivity, allowing the reaction to proceed in a more controlled manner [123].

**Fig. 15:**
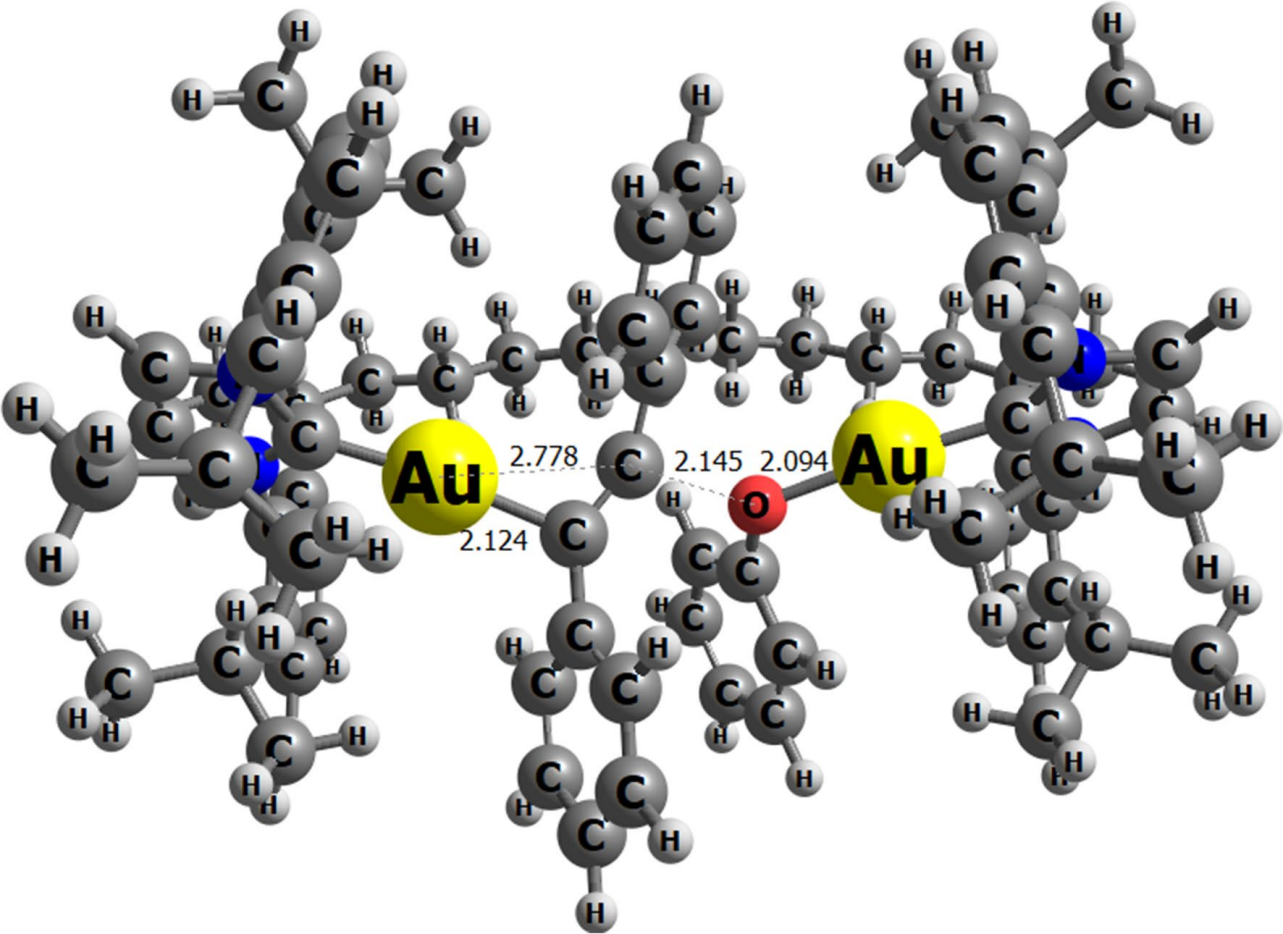
Transition state for the C-O bond formation with the diaurated catalyst, chelated by nine ethylene groups (distances in Å)

**Fig. 16:**
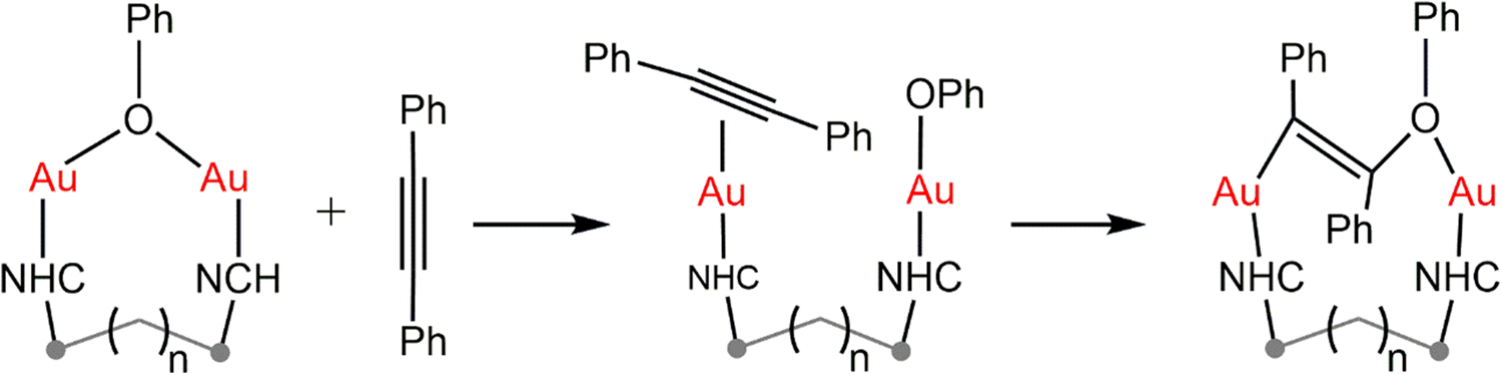
Intramolecular hydrophenoxylation scheme for the chelated digold complex

Poater and Cavallo, in collaboration with Nolan [124], transformed the intermolecular digold system into an intramolecular mechanism by DFT calculations (Fig. 15). They found that the breaking of the gem-digold hydroxide complex depended on the length of the ethylene chain (Fig. 16). Lower energy barriers and better thermodynamic profiles were observed with increased chain length, allowing for better accommodation of the metal moieties after the attack of PhOH or Ph-CC-Ph. However, while a longer chain provided more space and improved separation, it also introduced flexibility, potentially decreasing stability due to bending. Interestingly, PhOH was more effective in breaking the hydroxide dimer, showing superior kinetics and thermodynamics compared to the alkyne (Ph-CC-Ph). This preference was significant, considering the excess of the latter substrate and its smooth coordination leading to C-O bond formation.

Calculations demonstrated that chelation improves catalysis, supporting the encapsulation results of Nolan and Reek [122]. The rds in this intramolecular setup was identified as the protonation of the intermediate alkene by phenol, with a barrier of 34.8 kcal/mol over the initial complex.

## Conclusions

The historical trajectory of chemistry reveals the transformative impact of catalysis, with gold catalysis emerging as a significant milestone. Actually, the introduction of gold as a catalyst marked a pivotal evolution, extending its utility beyond traditional applications to play a crucial role in homogeneous catalysis.

Gold’s distinctive properties, including its strong electrophilicity, exceptional catalytic activity, and remarkable stability, have positioned it as a key element in the synthesis of complex molecules. Its high affinity for electron-rich species allows it to effectively activate substrates, facilitating a wide range of chemical transformations. Additionally, gold’s flexibility and resistance to oxidation make it a robust catalyst in various reaction environments, which is particularly advantageous in fields such as nanomedicine and green chemistry. Gold-based complexes have demonstrated notable efficiency in hydroalkoxylation and hydroamination reactions, where they promote the formation of carbon–oxygen bonds under mild conditions, thus minimizing energy consumption and reducing the need for harsh reagents. These attributes underline gold’s potential in advancing sustainable chemical processes and expanding its role in innovative applications, including the development of therapeutic agents and environmentally friendly synthesis methods.

Recent studies on dual gold catalysis and heterobimetallic complexes further underscore the versatility and efficacy of gold catalysts. These innovations highlight the potential for achieving high turnover rates and selectivity, propelling forward the scope of modern synthetic chemistry.

In summary, the evolution of gold catalysis exemplifies a crucial advancement in the field of chemistry. Its unique properties and the resulting innovations offer promising pathways for developing sustainable and efficient chemical processes. This progress not only enhances the theoretical understanding of catalysis but also paves the way for practical applications that could benefit various industries, from pharmaceuticals to environmental science. The continuous exploration and development of gold catalysis will likely remain a cornerstone of future chemical research and industrial application.

## Data Availability

No datasets were generated or analysed during the current study.
